# Heritability of Low ER Staining/HER2-Breast Tumors: Are We Missing an Opportunity for Germline Testing?

**DOI:** 10.3390/genes11121469

**Published:** 2020-12-08

**Authors:** Leann A. Lovejoy, Clesson E. Turner, Justin M. Wells, Craig D. Shriver, Rachel E. Ellsworth

**Affiliations:** 1 Chan Soon-Shiong Institute for Molecular Medicine at Windber, Windber, PA 15963, USA; l.lovejoy@wriwindber.org; 2 Department of Pediatrics, Uniformed Services University of the Health Sciences, Bethesda, MD 20814, USA; clesson.turner@usuhs.edu; 3 Department of Pathology, Walter Reed National Military Medical Center, Bethesda, MD 20814, USA; justin.m.wells8.civ@mail.mil; 4 Department of Surgery, Uniformed Services University of the Health Sciences, Bethesda, MD 20814, USA; craig.shriver@usuhs.edu; 5 Murtha Cancer Center/Research Program, Uniformed Services University of the Health Sciences and Walter Reed National Military Medical Center, Bethesda, MD 20814, USA; 6 Henry M. Jackson Foundation for the Advancement of Military Medicine, Inc., Windber, PA 15963, USA

**Keywords:** genetic testing, breast cancer, ER low positive, ASCO/CAP guidelines

## Abstract

In 2010, the genetic testing criteria was changed to allow women diagnosed ≤ 60 years old with triple negative breast cancer (TNBC) to undergo germline testing. In the same year, estrogen receptor (ER) positivity was defined as having ≥1% ER staining cells. While tumors with 1–10% ER staining cells and HER2 negative (HER2-) status share characteristics with TNBC, the utility of germline testing in women with ER low positive/HER2- (ERLP/HER2-) tumors is not well-understood. To this end, all patients with hormone receptor positive staining cells ≤ 10% and negative HER2 status were identified. Clinical genetic test results were extracted for patients who underwent testing. Panel testing was performed for those women who had genomic DNA available for research purposes. ERLP/HER2-tumors constituted 2.7% of all tumors in the database. Patients did not differ significantly from those with TNBC by age at diagnosis, ethnicity, family history or tumor size, stage or grade (*p* > 0.05). Mutation frequency did not differ significantly (*p* = 0.757) between groups (ERLP/HER2- 16.1%; TNBC 16.7%). Hereditary forms of breast cancer were similar in both ERLP/HER2- and TNBC, thus current guidelines may result in the under testing of women with low ER tumors, resulting in missed opportunities to improve patient management.

## 1. Introduction

Triple negative breast cancers (TNBC), do not express estrogen receptors (ERs), progesterone receptors (PRs), or HER2, and are associated with a poor prognosis [1]. Germline mutations in the *BRCA1* and *BRCA2* genes have been detected in 15% of patients with TNBC [2]. Women with germline mutations in the *BRCA* genes and TNBC may be sensitive to platinum agents [3]. In addition, Poly ADP Ribose Polymerase (PARP) inhibitors have been approved by the FDA for the treatment of TNBC in patients with germline *BRCA* mutations [4].

In 2010, two events altered genetic testing eligibility for women with invasive breast cancer. First, the National Comprehensive Cancer Network (NCCN) included TNBC in their guidelines for germline testing of *BRCA* genes, thus expanding the number of women eligible for testing [5]. Second, the American Society of Clinical Oncology (ASCO) and College of American Pathologists (CAP) recommended that a cutoff of 1% positive cells should be used to define ER and PR positive status [6]. Using these criteria, only HER2 negative (HER2-) tumors with <1% hormone receptor staining cells would be considered TNBC, precluding testing for women with low levels of hormone receptor and HER2-tumors who do not otherwise meet test eligibility criteria. Gene expression studies, however, have demonstrated that the majority of tumors with 1–9% ER staining have ER negative intrinsic subtypes [7,8], and patients with low-staining tumors have survival rates similar to those with ER negative tumors [9]. The updated ASCO/CAP guidelines published in 2020 recommended that tumors with 1–10% ER positive cells should be classified as ER low positive (ERLP) [10].

Although ASCO/CAP has recognized that tumors with low ER staining should be recognized as a special type of tumor, the cutoff for defining ER positivity remains ≥ 1%. Under this criterion, ERLP tumors that are HER2 negative would not be classified as TNBC, potentially causing some women to miss the opportunity for clinical genetic testing. In this study, we performed panel testing in patients with ERLP and HER2 negative (ERLP/HER2-) tumors or TNBC to determine whether the germline mutation frequency and array of mutations in women with ERLP/HER2-tumors is similar to those in women with TNBC. The results suggest that ERLP/HER2-tumors should be included in the NCCN test eligibility criteria.

## 2. Materials and Methods

Patients from the Walter Reed National Military Medical Center (WRNMMC), Bethesda, MD, USA; the Anne Arundel Medical Center, Annapolis, MD, USA; or the Joyce Murtha Breast Cancer Center, Windber, PA, USA, were enrolled in the Clinical Breast Care Project (CBCP) starting in 2001. As both NCCN and ASCO/CAP guidelines were issued in 2010, only patients diagnosed 2010 through 2019 were included in this study. Patients were eligible for enrollment if they were ≥18 years of age and were mentally competent and willing to provide informed consent. This work was performed with approval from the WRNMMC Human Use Committee and Institutional Review Board (protocol WRNMMC IRB #20704).

Pathological characterization was performed as previously described [7]. ER and PR status were determined by IHC analysis, and the percentage of stained cells were recorded in the CBCP database. HER2 status was defined using ASCO/CAP guidelines [11]. Family history was classified by the number of first- or second-degree family members with breast, ovarian, or pancreatic cancer. Test eligibility was assigned using the NCCN Genetic/Familial High-risk Assessment: Breast, Ovarian, and Pancreatic guidelines version 1.2020 (genetics_bop.pdf (nccn.org)). The test results were extracted from the CBCP database for the 161 patients who underwent clinical genetic testing.

Blood samples were available from 117 women who did not undergo genetic testing. Genomic DNA was isolated from blood clots using the Gentra Clotspin and Puregene DNA purification kits (Qiagen, Valencia, CA, USA). Isolated DNA was quantitated using the Qubit^®^ Fluorometer (Thermo Fisher Scientific, Waltham, MA, USA). To generate the sequencing libraries, 50 ng of DNA was processed using the Illumina DNA prep with enrichment kit and TruSight Cancer panel (Illumina, Inc, San Diego, CA, USA). The resulting libraries were sequenced on a MiSeq (Illumina, Inc, San Diego, CA, USA). Variant Interpreter (Illumina, Inc, San Diego, CA, USA) was used for the data analysis; only variants with a minor allele frequency of >25% were included [12]. Variants were classified using the ClinVar database (http://www.clinvar.com/). Statistical analyses were performed using Fisher’s exact tests or Chi-square analyses, with significance defined by *p* < 0.05.

## 3. Results

### 3.1. Patient Characteristics

**Figure 1:**
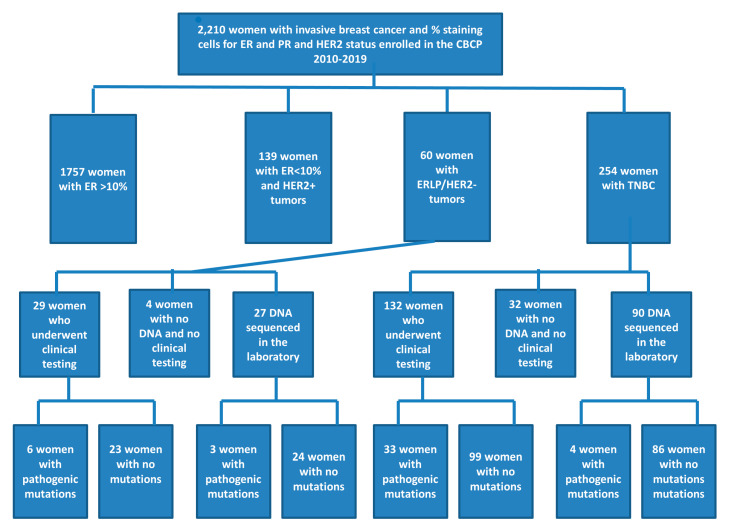
Flow-chart describing biomarker status, test uptake, and sequencing results.

Of the 2210 women with invasive breast cancer who enrolled in the CBCP between 2010 and 2019, 60 (2.7%) had ERLP/HER2-tumors with PR staining ≤ 10% tumors and 254 (11.5%) had TNBC (Figure 1). The average age at diagnosis was 57.3 years old in women with ERLP/HER2-tumors and 56.5 years in those with TNBC (*p* = 0.700). Ethnicity; family history; and tumor size, stage, and grade did not differ significantly between groups (Table 1).

### 3.2. Test Eligibility and Uptake

Significantly fewer (*p* = 0.015) women with ERLP/HER2- (67.7%) were eligible for genetic testing compared with those with TNBC (81.1%). The test uptake did not differ significantly (*p* = 0.305) in the two groups with 29/40 (67.5%) women with ERLP/HER2- and 132/206 (61.2%) women with TNBC pursuing clinical genetic testing.

### 3.3. Genetic Results

**Figure 2:**
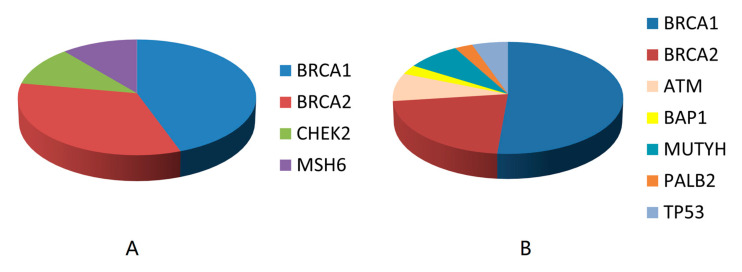
Frequency of mutations by genes in ERLP/HER2-tumors (**A**) and TNBC (**B**).

Germline data were available for 56/60 women with ERLP/HER2-tumors and 222/254 women with TNBC. The mutation frequency did not differ significantly (*p* = 0.757) between groups (ERLP/HER2- 16.1%; TNBC 16.7%). Mutations were detected in four genes, *BRCA1* (*n* = 4), *BRCA2* (*n* = 3), *CHEK2* (*n* = 1), and *MSH6* (*n* = 1) in women with ERLP/HER2-, and in seven genes, *ATM* (*n* = 3), *BAP1* (*n* = 1), *BRCA1* (*n* = 20), *BRCA2* (*n* = 8), *MUTYH* (*n* = 2), *PALB2* (*n* = 1), and *TP53* (*n* = 2), in women with TNBC (Table 2). Mutations in *BRCA1* were the most common in both groups (44.4% ERLP/HER2- and 54.1% TNBC; Figure 2).

### 3.4. Mutation Frequency in Women Not Eligible for Genetic Testing

Eleven (18.3%) women diagnosed between 45 and 60 years of age with ERLP/HER2- did not have a significant family history of breast cancer. One (9.1%) of these test-ineligible women harbored a germline mutation in BRCA2, with tumor biomarkers reported as 10% ER, 1% PR, and 0+ HER2. Within the group of 46 women diagnosed with TNBC between 45 and 60 years of age but without a significant family history of breast cancer, three (6.5%) harbored mutations in ATM (*n* = 1), BAP1 (*n* = 1), or BRCA2 (*n* = 1). The mutation frequency was not significantly different (*p* = 0.825) between these two groups of women.

## 4. Discussion

Since the identification of the *BRCA1* and *BRCA2* genes over 25 years ago [13,14], the benefits of germline testing have expanded to include not only personal and familial risk assessment, but also treatment choice. For women with *BRCA* germline mutations, patients may elect contralateral prophylactic mastectomy and oophorectomy [15]. In patients with TNBC and BRCA mutations, platinum salts and PARP inhibitors, which exploit the genomic instability of cells with defective DNA repair, may be beneficial [16]. Surveillance and surgical strategies have also been developed for women with germline mutations in non-BRCA genes in order to prevent or facilitate in the early detection of additional cancers [17]. The opportunity to utilize these precision medicine approaches may not be available for women who harbor germline mutations but that are ineligible for germline testing using the current criteria.

Within our dataset, the mutation frequency and array of mutated genes did not differ significantly between women with TNBC and ERLP/HER2-tumors, suggesting that germline testing would be beneficial in women with ER low-staining tumors. Of note, under the current ASCO/CAP guidelines, which define ER tumor staining of 1–10% as ER positive, 18.3% of women in the ERLP/HER2- group diagnosed at 46–60 years of age without a family history, were not eligible for genetic testing, while the 18.1% of women with TNBC with similar characteristics would be eligible. Moreover, the mutation frequency within this test-ineligible group did not differ significantly from their counterparts with TNBC. Nine percent of these test-ineligible women may have benefited from precision medicine approaches afforded by the determination of the mutational background.

To our knowledge, one other publication examined germline mutations in low ER staining/HER2-breast tumors. A study from The University of Texas MD Anderson Cancer Center evaluated mutation frequencies in 314 patients with HER2-ER < 10% tumors with *BRCA1/2* genetic test results. That study found that 238 women had TNBC and 76 women had hormone receptor (HR) 1–9%/HER2-tumors [18]. As in our study, mutation frequencies did not differ significantly between patients with TNBC and those with HR-low positive tumors. The *BRCA* mutation frequency in the MD Anderson study (36.9%) was, however, significantly higher (*p* < 0.001) than that detected in our study (16.9%). This difference is likely attributable to the following inclusion criteria: all of the patients in the MD Anderson study were referred for genetic counseling between 2004 and 2014, while our study included all of the patients diagnosed between 2010–2020 with HER2-/ER ≤ 10% tumors, 21.6% of whom were not eligible for genetic testing using the NCCN 1.2020 criteria. In addition, our study evaluated a more extensive (*n* = 94) set of cancer predisposition genes, and detected mutations in genes such as *PALB2,* which has been associated with heritable forms of TNBC [19]. Despite these differences, both data sets suggest that the ER ≥ 1% threshold defined by ASCO/CAP may lead to under testing.

This study has several limitations. While the frequency of ERLP/HER2-tumors was within the 2–3% range reported by ASCO/CAP [10], only 60 cases were reported in the past decade. Of these, four women had no germline testing and no DNA available for research purposes, which may have influenced the mutation frequency. In addition, data do not yet exist as to whether women with ERLP/HER2-tumors, especially those diagnosed at 46–60 years old without a significant family history, would pursue genetic testing or, for those with *BRCA* mutations, would benefit from PARP inhibitors or platinum agents.

## 5. Conclusions

These data demonstrate that women with ERLP/HER2-tumors have germline mutation frequencies similar to those detected in women with TNBC, with *BRCA1* and *BRCA2* being the most frequently mutated genes in this population. These data suggest that while ASCO/CAP guidelines classify tumors with ER staining 1–10% as ER positive, women with ERLP/HER2-tumors may benefit from germline genetic testing to improve patient management and provide the opportunity for family planning and risk assessment.

## Figures and Tables

**Table 1:** Demographic and pathologic characteristics of women with estrogen receptor low positive (ERLP)/ HER2 negative (HER2-) and triple negative breast cancers (TNBC) tumors.

Characteristic	ERLPHN (*n* = 60)	TNBC (*n* = 254)	*p*-Value
Age			0.443
≤45 years	11 (18.3%)	55 (21.7%)	
46–60 years	30 (50.0%)	104 (40.9%)	
>60 years	19 (31.7%)	95 (37.4%)	
Ethnicity			0.178
African American	13 (21.6%)	85 (33.4)	
Asian	2 (3.3%)	5 (2.0%)	
Hispanic	1 (1.7%)	6 (2.4%)	
Other	1 (1.7%)	3 (1.2%)	
European American	42 (70.0%)	155 (61.0%)	
Unknown	1 (1.7%)	0 (0.0%)	
Family history			0.197
0	18 (30.0%)	108 (42.5%)	
1	25 (41.7%)	78 (30.7%)	
2	9 (15.0%)	46 (18.1%)	
≥3	8 (13.3%)	20 (7.9%)	
Unknown	0 (0.0%)	2 (0.8%)	
Tumor size			0.189
T1	22 (36.7%)	131 (51.6%)	
T2	30 (50.0%)	97 (38.2%)	
T3/4	6 (10.0%)	22 (8.6%)	
Unknown	2 (3.3%)	4 (1.6%)	
Tumor grade			0.488
Well-differentiated	2 (3.3%)	4 (1.6%)	
Moderately-differentiated	12 (20.0%)	35 (13.8%)	
Poorly-differentiated	45 (75.0%)	211 (83.0%)	
Unknown	1 (1.7%)	4 (1.6%)	
Tumor stage			0.092
I	18 (30.0%)	109 (42.9%)	
II	30 (50.0%)	111 (43.7%)	
III	9 (15.0%)	31 (12.2%)	
IV	3 (5.0%)	3 (1.2%)	

**Table 2:** Mutations within women with ERLP/HER2-tumors (*n* = 9) or TNBC (*n* = 37).

Patient	Gene	Mutation	≤45 Years at Diagnosis	<60 Years at Diagnosis	Family History ^a^
**ERLP/HER2-**					
38	*BRCA1*	Glu1250Ter	√	√	√
185	*BRCA1*	Mutation not provided ^b^		√	√
191	*BRCA1*	Glu1250Ter	√	√	√
226	*BRCA1*	Gln1756Profs	√	√	√
78	*BRCA2*	Leu1466Phefs			√
117	*BRCA2*	Leu2510Pro		√	
125	*BRCA2*	Ile1859Lysfs		√	√
171	*CHEK2*	Thr367Metfs			√
213	*MSH6*	Phe1088Leufs		√	√
**TNBC**					
280	*ATM*	Glu2236Ter			
230	*ATM*	901 + 1G > A		√	
289	*ATM*	Tyr1124Ter		√	
303	*BAP1*	Leu573fs		√	
5	*BRCA1*	5193 + 2del			√
48	*BRCA1*	Gln1408Ter		√	√
54	*BRCA1*	Met1775Arg		√	√
73	*BRCA1*	5467 + 1G > A	√	√	
80	*BRCA1*	Glu908Ter	√		
110	*BRCA1*	Gln1756Profs		√	
112	*BRCA1*	Ser1655fs	√	√	√
123	*BRCA1*	4986 + 6T > G	√	√	√
150	*BRCA1*	Val1234fs	√	√	√
159	*BRCA1*	Val1688del	√	√	
172	*BRCA1*	4986 + 3G > C	√	√	
182	*BRCA1*	Glu23fs	√	√	
201	*BRCA1*	Mutation not provided ^b^	√	√	√
219	*BRCA1*	Val1838Glu	√	√	√
237	*BRCA1*	Glu23fs	√	√	
239	*BRCA1*	Mutation not provided ^b^	√		
280	*BRCA1*	Met1775Arg			
283	*BRCA1*	135-1G > T	√	√	√
288	*BRCA1*	Thr276fs	√	√	
310	*BRCA1*	Lys894fs	√	√	√
62	*BRCA2*	Asp1199_Cys1200insTer			
109	*BRCA2*	Tyr1894Terfs		√	√
139	*BRCA2*	Asn1784fs			√
236	*BRCA2*	Ile2315fs			
238	*BRCA2*	Asn1626Serfs			
268	*BRCA2*	Asn986fs			√
277	*BRCA2*	Leu2805fs		√	√
308	*BRCA2*	Cys1200Terfs		√	
133	*MUTYH*	Arg233Ter		√	
221	*MUTYH*	850-2A > G		√	√
241	*PALB2*	Tyr1183Ter		√	√
220	*TP53*	Arg175Gly		√	√
300	*TP53*	Arg273Cys	√	√	

^a^ Family history defined using National Comprehensive Cancer Network (NCCN) 1.2020 criteria for determining hereditary testing eligibility. ^b^ for three patients, the exact mutation from clinical testing was not provided in the database.
